# *exo*-1,7-Dimethyl-4-phenyl-10-oxa-4-aza­tri­cyclo­[5.2.1.0^2,6^]dec-8-ene-3,5-dione

**DOI:** 10.1107/S1600536813030262

**Published:** 2013-11-09

**Authors:** Armando Pineda-Contreras, Oscar Fernando Vázquez-Vuelvas, Laura Edith Negrete-López, Héctor García-Ortega, Marcos Flores-Alamo

**Affiliations:** a Facultad de Ciencias Químicas, Universidad de Colima, Km 9 Carretera Colima-Coquimatlán, Apartado Postal 29000, Coquimatlán, Colima, Mexico; b Facultad de Química, Universidad Nacional Autónoma de México, Ciudad Universitaria, 04510, México D.F., Mexico

## Abstract

The title compound, C_16_H_15_NO_3_, consists of an oxabicycle fused to an *N*-phenyl-substituted pyrrolidine ring *anti* to the double bond, affording the *exo* isomer. In the oxabicycle system, the six-membered ring presents a boat conformation, while the heterocyclic rings show envelope conformations with the O atom projected out of the plane. In the crystal, adjacent mol­ecules are linked *via* weak C—H⋯O hydrogen bonds, forming chains propagating along the *a*-axis direction. The chains are linked by C—H⋯π inter­actions, forming two-dimensional networks lying parallel to the *ac* plane.

## Related literature

Monomeric norbornene derivatives synthesized by Diels–Alder reactions have attracted great attention due to the attractive optical, thermal, and electrochemical properties of the resulting polymers, see: Choi *et al.* (2010 ▶); Khosravi & Al-Hajaji (1998 ▶). For related structures, see: Li (2010 ▶, 2011 ▶); Jarosz *et al.* (2001 ▶).
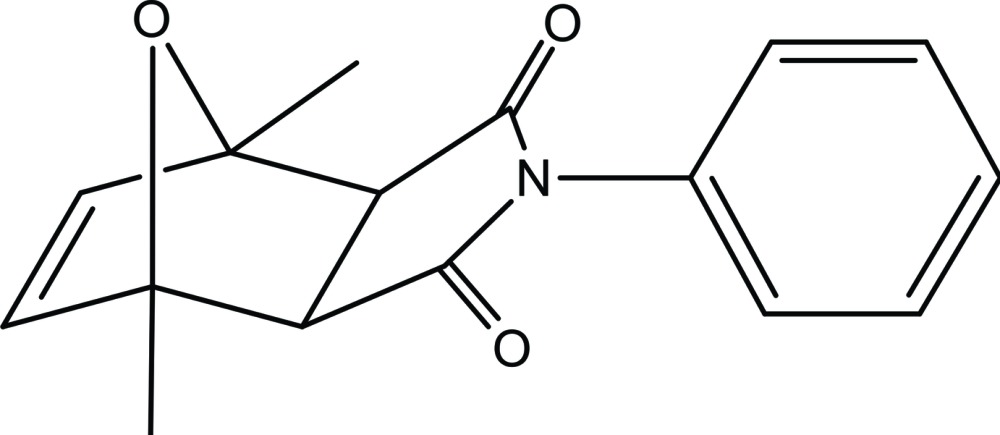


## Experimental

### 

#### Crystal data

C_16_H_15_NO_3_
*M* *_r_* = 269.29
Monoclinic, 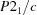

*a* = 8.1267 (6) Å
*b* = 9.8570 (8) Å
*c* = 17.2099 (12) Å
β = 93.564 (7)°
*V* = 1375.93 (18) Å^3^
*Z* = 4
Mo *K*α radiation
μ = 0.09 mm^−1^
*T* = 298 K
0.58 × 0.54 × 0.22 mm

#### Data collection

Agilent Xcalibur (Atlas, Gemini) diffractometer
Absorption correction: analytical (*CrysAlis PRO*; Agilent, 2011 ▶) *T*_min_ = 0.958, *T*_max_ = 0.983
6009 measured reflections
2709 independent reflections
2104 reflections with *I* > 2σ(*I*)
*R*_int_ = 0.019

#### Refinement

*R*[*F* ^2^ > 2σ(*F* ^2^)] = 0.040
*wR*(*F* ^2^) = 0.101
*S* = 1.02
2709 reflections
184 parameters
H-atom parameters constrained
Δρ_max_ = 0.24 e Å^−3^
Δρ_min_ = −0.16 e Å^−3^

### 

Data collection: *CrysAlis PRO* (Agilent, 2011 ▶); cell refinement: *CrysAlis PRO*; data reduction: *CrysAlis PRO*; program(s) used to solve structure: *SHELXS97* (Sheldrick, 2008 ▶); program(s) used to refine structure: *SHELXL97* (Sheldrick, 2008 ▶); molecular graphics: *ORTEP-3 for Windows* (Farrugia, 2012 ▶) and *Mercury* (Macrae *et al.*, 2008 ▶); software used to prepare material for publication: *WinGX* (Farrugia, 2012 ▶).

## Supplementary Material

Crystal structure: contains datablock(s) global, I. DOI: 10.1107/S1600536813030262/bh2487sup1.cif

Structure factors: contains datablock(s) I. DOI: 10.1107/S1600536813030262/bh2487Isup2.hkl

Supplementary material file. DOI: 10.1107/S1600536813030262/bh2487Isup3.cml

Additional supplementary materials:  crystallographic information; 3D view; checkCIF report

## Figures and Tables

**Table 1:** Hydrogen-bond geometry (Å, °) *Cg*1 is the centroid of the C15–C20 phenyl ring.

*D*—H⋯*A*	*D*—H	H⋯*A*	*D*⋯*A*	*D*—H⋯*A*
C5—H5⋯O14^i^	0.93	2.57	3.362 (2)	144
C10—H10*B*⋯*Cg*1^i^	0.96	2.95	3.8029	148
C12—H12*B*⋯*Cg*1^ii^	0.96	2.79	3.7287	167

Symmetry codes: (i) 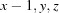; (ii) 
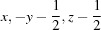
.

**Fig. 1.**
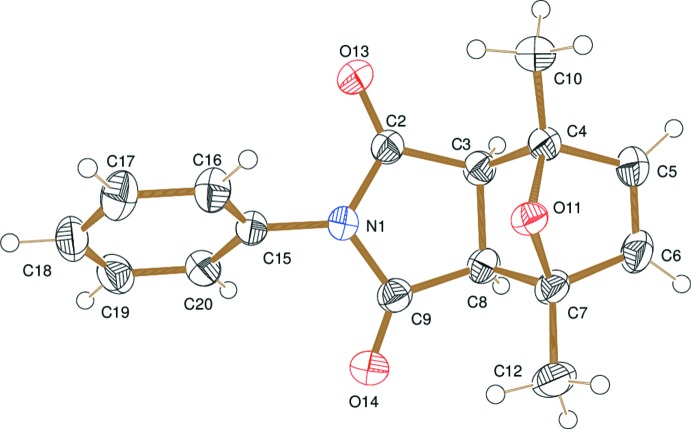
Molecular structure with displacement ellipsoids for non-H atoms drawn at the 40% probability level.

**Fig. 2.**
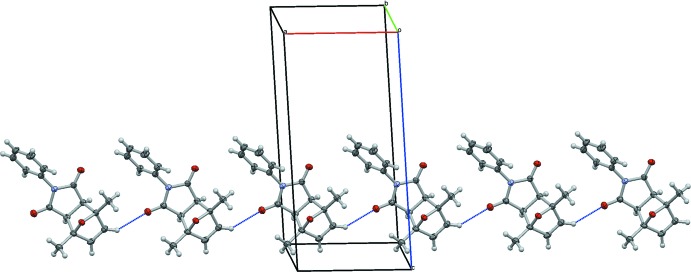
Weak intermolecular hydrogen bond of the title compound viewed down the b axis. The weak C—H···O interactions are showed as dashed lines.

## supplementary crystallographic information

### 1. Comment

Diels-Alder reaction is used to synthesize 5-norbornene-2,3-dicarboximide by reaction between cyclopentadiene and maleic anhydride, followed by imidization with a primary amine (Choi *et al.*, 2010; Khosravi & Al-Hajaji, 1998). However the Diels-Alder adduct is predominantly the *endo* isomer. In this regard, the use of furan derivatives instead of cyclopentadiene affords the *exo* isomer at very high yield (Li, 2010, 2011; Jarosz *et al.*, 2001).

X-ray crystallography confirmed the molecular structure and the atom connectivity for the title compound, as illustrated in Fig. 1. The oxabicycle moiety is bound *exo* with respect to the *N*-maleimide group. This five-membered imide cycle shows a planar geometry by the contribution of the two carbonyl groups and the *sp*^2^ hybridized nitrogen atom N1. This is a consequence of the conjugative delocalization of the nitrogen lone pair, and is supported by the sum of C—N—C angles around N1 of 359.8 (18)°. The phenyl ring (C15···C20) presents a rotation with respect of the plane of the maleimide group, indicated by the torsion angle C20—C15—N1—C2, which is 118.53 (17)°.

Weak hydrogen bonds stabilize the crystal packing by the presence of C—H···O and C—H···π interactions, which are listed in the Table 1. The intermolecular C—H···π contacts involve the C10—H10B···*Cg1* and C12—H12B···*Cg1* (*Cg1* is the centroid of the phenyl ring C15···C20). Moreover, the weak C—H···O interaction formed by C5—H5···O14 propagates along the *ac* plane (Fig. 2).

### 2. Experimental

Diels-Alder adduct was obtained by the reaction between 2,5-dimethylfuran (0.5 g, 5.2 mmol) and *N*-phenylmaleimide (1.0 g, 5.7 mmol) in ethyl acetate (7.5 ml). The mixture was stirred for 16 h at 333 K. After cooling, the formed precipitate was removed by filtration under vacuum. The collected filtrate was recrystallized twice from ethyl acetate. The title compound was obtained in 86% yield, m.p. 401–403 K. The single-crystal suitable for X-ray determination was obtained by evaporation of an ethyl acetate solution of the title compound over 5 days.

### 3. Refinement

All H atoms were placed in geometrical idealized positions and were refined as riding on their parent atoms, with C—H = 0.93–0.98 Å, and with *U*_iso_(H) = 1.2 *U*_eq_(CH) or 1.5 *U*_eq_(CH_3_).

### Crystal data

**Table:**

C_16_H_15_NO_3_	*D*_x_ = 1.3 Mg m^−^^3^
*M**_r_* = 269.29	Melting point: 401 K
Monoclinic, *P*2_1_/*c*	Mo *K*α radiation, λ = 0.71073 Å
*a* = 8.1267 (6) Å	Cell parameters from 1980 reflections
*b* = 9.8570 (8) Å	θ = 3.4–26.0°
*c* = 17.2099 (12) Å	µ = 0.09 mm^−^^1^
β = 93.564 (7)°	*T* = 298 K
*V* = 1375.93 (18) Å^3^	Block, colourless
*Z* = 4	0.58 × 0.54 × 0.22 mm
*F*(000) = 568	

### Data collection

**Table:**

Agilent Xcalibur (Atlas, Gemini) diffractometer	2709 independent reflections
Graphite monochromator	2104 reflections with *I* > 2σ(*I*)
Detector resolution: 10.4685 pixels mm^-1^	*R*_int_ = 0.019
ω scans	θ_max_ = 26.1°, θ_min_ = 3.4°
Absorption correction: analytical (*CrysAlis PRO*; Agilent, 2011)	*h* = −10→9
*T*_min_ = 0.958, *T*_max_ = 0.983	*k* = −12→12
6009 measured reflections	*l* = −21→19

### Refinement

**Table:**

Refinement on *F*^2^	Secondary atom site location: difference Fourier map
Least-squares matrix: full	Hydrogen site location: inferred from neighbouring sites
*R*[*F*^2^ > 2σ(*F*^2^)] = 0.040	H-atom parameters constrained
*wR*(*F*^2^) = 0.101	*w* = 1/[σ^2^(*F*_o_^2^) + (0.0389*P*)^2^ + 0.4058*P*] where *P* = (*F*_o_^2^ + 2*F*_c_^2^)/3
*S* = 1.02	(Δ/σ)_max_ = 0.001
2709 reflections	Δρ_max_ = 0.24 e Å^−^^3^
184 parameters	Δρ_min_ = −0.16 e Å^−^^3^
0 restraints	Extinction correction: *SHELXL97* (Sheldrick, 2008), Fc^*^=kFc[1+0.001xFc^2^λ^3^/sin(2θ)]^-1/4^
0 constraints	Extinction coefficient: 0.048 (3)
Primary atom site location: structure-invariant direct methods	

### Special details

**Table:**

Experimental. Absorption correction: (CrysAlis PRO; Agilent, 2011) Analytical numeric absorption correction using a multifaceted crystal model

### Fractional atomic coordinates and isotropic or equivalent isotropic displacement parameters (Å^2^)

**Table:**

	*x*	*y*	*z*	*U*_iso_*/*U*_eq_	
C2	0.89127 (19)	0.24866 (16)	0.65338 (8)	0.0379 (4)	
C3	0.81574 (19)	0.27178 (15)	0.73016 (8)	0.0375 (4)	
H3	0.7482	0.3542	0.7296	0.045*	
C4	0.72028 (18)	0.14469 (16)	0.75948 (8)	0.0372 (4)	
C5	0.6482 (2)	0.19228 (18)	0.83406 (9)	0.0472 (4)	
H5	0.5382	0.2114	0.8413	0.057*	
C6	0.7726 (2)	0.20099 (18)	0.88640 (9)	0.0484 (4)	
H6	0.7676	0.2279	0.938	0.058*	
C7	0.92630 (19)	0.15872 (16)	0.84656 (8)	0.0386 (4)	
C8	0.96335 (19)	0.27970 (16)	0.79084 (8)	0.0379 (4)	
H8	0.9688	0.367	0.8181	0.045*	
C9	1.1131 (2)	0.25456 (16)	0.74554 (9)	0.0393 (4)	
C10	0.6092 (2)	0.06732 (19)	0.70213 (9)	0.0486 (4)	
H10A	0.6726	0.033	0.6612	0.073*	
H10B	0.5245	0.1265	0.6805	0.073*	
H10C	0.5597	−0.007	0.7281	0.073*	
C12	1.0660 (2)	0.0990 (2)	0.89652 (9)	0.0533 (5)	
H12A	1.027	0.022	0.9242	0.08*	
H12B	1.1084	0.1658	0.933	0.08*	
H12C	1.1519	0.0708	0.8642	0.08*	
C15	1.17344 (18)	0.21261 (16)	0.60751 (8)	0.0377 (4)	
C16	1.1598 (2)	0.09518 (17)	0.56431 (9)	0.0478 (4)	
H16	1.078	0.0322	0.5734	0.057*	
C17	1.2689 (2)	0.07181 (19)	0.50718 (10)	0.0556 (5)	
H17	1.2603	−0.0072	0.4776	0.067*	
C18	1.3896 (2)	0.1645 (2)	0.49394 (10)	0.0543 (5)	
H18	1.4629	0.1482	0.4556	0.065*	
C19	1.4025 (2)	0.2812 (2)	0.53705 (10)	0.0521 (5)	
H19	1.4848	0.3437	0.528	0.063*	
C20	1.29349 (19)	0.30661 (18)	0.59406 (9)	0.0442 (4)	
H20	1.3014	0.3863	0.6229	0.053*	
O11	0.85844 (12)	0.06324 (10)	0.78944 (5)	0.0362 (3)	
O13	0.82053 (14)	0.24123 (13)	0.58982 (6)	0.0525 (3)	
O14	1.25472 (14)	0.24824 (14)	0.77023 (7)	0.0556 (4)	
N1	1.06138 (15)	0.23679 (13)	0.66731 (7)	0.0381 (3)	

### Atomic displacement parameters (Å^2^)

**Table:**

	*U*^11^	*U*^22^	*U*^33^	*U*^12^	*U*^13^	*U*^23^
C2	0.0386 (8)	0.0413 (8)	0.0339 (8)	0.0014 (7)	0.0028 (6)	0.0026 (7)
C3	0.0374 (8)	0.0387 (8)	0.0365 (8)	0.0073 (7)	0.0044 (6)	−0.0010 (6)
C4	0.0337 (8)	0.0440 (9)	0.0340 (7)	0.0042 (7)	0.0029 (6)	0.0002 (7)
C5	0.0426 (9)	0.0590 (10)	0.0413 (9)	0.0037 (8)	0.0123 (7)	−0.0020 (8)
C6	0.0583 (11)	0.0552 (10)	0.0332 (8)	−0.0037 (9)	0.0136 (7)	−0.0068 (7)
C7	0.0443 (9)	0.0430 (9)	0.0284 (7)	−0.0022 (7)	0.0001 (6)	−0.0049 (6)
C8	0.0444 (9)	0.0363 (8)	0.0332 (7)	0.0005 (7)	0.0041 (6)	−0.0068 (6)
C9	0.0407 (9)	0.0407 (8)	0.0363 (8)	−0.0026 (7)	0.0006 (7)	−0.0007 (7)
C10	0.0407 (9)	0.0615 (11)	0.0430 (9)	−0.0035 (8)	−0.0035 (7)	−0.0006 (8)
C12	0.0558 (11)	0.0633 (12)	0.0390 (9)	−0.0051 (9)	−0.0111 (8)	0.0054 (8)
C15	0.0360 (8)	0.0449 (9)	0.0326 (7)	0.0037 (7)	0.0046 (6)	0.0018 (7)
C16	0.0544 (10)	0.0455 (9)	0.0448 (9)	−0.0038 (8)	0.0129 (8)	−0.0010 (8)
C17	0.0689 (12)	0.0526 (11)	0.0469 (10)	0.0084 (10)	0.0167 (9)	−0.0054 (8)
C18	0.0504 (11)	0.0691 (13)	0.0454 (9)	0.0155 (9)	0.0176 (8)	0.0068 (9)
C19	0.0401 (9)	0.0626 (12)	0.0544 (10)	−0.0001 (8)	0.0095 (8)	0.0137 (9)
C20	0.0397 (9)	0.0474 (9)	0.0457 (9)	−0.0005 (8)	0.0038 (7)	0.0006 (8)
O11	0.0379 (6)	0.0376 (6)	0.0327 (5)	0.0011 (5)	−0.0021 (4)	−0.0027 (4)
O13	0.0456 (7)	0.0792 (9)	0.0323 (6)	0.0025 (6)	−0.0016 (5)	0.0041 (6)
O14	0.0395 (7)	0.0805 (9)	0.0462 (7)	−0.0041 (6)	−0.0029 (5)	0.0001 (6)
N1	0.0358 (7)	0.0472 (8)	0.0317 (6)	0.0004 (6)	0.0051 (5)	−0.0022 (5)

### Geometric parameters (Å, º)

**Table:**

C2—O13	1.2059 (18)	C9—N1	1.3962 (19)
C2—N1	1.3932 (19)	C10—H10A	0.96
C2—C3	1.508 (2)	C10—H10B	0.96
C3—C8	1.543 (2)	C10—H10C	0.96
C3—C4	1.573 (2)	C12—H12A	0.96
C3—H3	0.98	C12—H12B	0.96
C4—O11	1.4488 (17)	C12—H12C	0.96
C4—C10	1.503 (2)	C15—C20	1.375 (2)
C4—C5	1.518 (2)	C15—C16	1.376 (2)
C5—C6	1.314 (2)	C15—N1	1.4359 (18)
C5—H5	0.93	C16—C17	1.384 (2)
C6—C7	1.520 (2)	C16—H16	0.93
C6—H6	0.93	C17—C18	1.370 (3)
C7—O11	1.4454 (17)	C17—H17	0.93
C7—C12	1.501 (2)	C18—C19	1.369 (3)
C7—C8	1.571 (2)	C18—H18	0.93
C8—C9	1.506 (2)	C19—C20	1.385 (2)
C8—H8	0.98	C19—H19	0.93
C9—O14	1.2036 (19)	C20—H20	0.93
			
O13—C2—N1	124.26 (14)	N1—C9—C8	108.39 (13)
O13—C2—C3	127.35 (14)	C4—C10—H10A	109.5
N1—C2—C3	108.39 (12)	C4—C10—H10B	109.5
C2—C3—C8	105.01 (12)	H10A—C10—H10B	109.5
C2—C3—C4	113.35 (12)	C4—C10—H10C	109.5
C8—C3—C4	101.62 (11)	H10A—C10—H10C	109.5
C2—C3—H3	112.1	H10B—C10—H10C	109.5
C8—C3—H3	112.1	C7—C12—H12A	109.5
C4—C3—H3	112.1	C7—C12—H12B	109.5
O11—C4—C10	111.89 (13)	H12A—C12—H12B	109.5
O11—C4—C5	101.62 (11)	C7—C12—H12C	109.5
C10—C4—C5	117.68 (13)	H12A—C12—H12C	109.5
O11—C4—C3	99.72 (11)	H12B—C12—H12C	109.5
C10—C4—C3	118.77 (12)	C20—C15—C16	120.66 (14)
C5—C4—C3	104.42 (13)	C20—C15—N1	119.79 (14)
C6—C5—C4	106.20 (14)	C16—C15—N1	119.55 (14)
C6—C5—H5	126.9	C15—C16—C17	119.34 (16)
C4—C5—H5	126.9	C15—C16—H16	120.3
C5—C6—C7	106.95 (14)	C17—C16—H16	120.3
C5—C6—H6	126.5	C18—C17—C16	120.28 (17)
C7—C6—H6	126.5	C18—C17—H17	119.9
O11—C7—C12	112.15 (13)	C16—C17—H17	119.9
O11—C7—C6	101.30 (12)	C19—C18—C17	120.12 (16)
C12—C7—C6	117.55 (13)	C19—C18—H18	119.9
O11—C7—C8	99.13 (11)	C17—C18—H18	119.9
C12—C7—C8	118.73 (14)	C18—C19—C20	120.32 (17)
C6—C7—C8	105.15 (13)	C18—C19—H19	119.8
C9—C8—C3	105.10 (12)	C20—C19—H19	119.8
C9—C8—C7	112.51 (13)	C15—C20—C19	119.28 (16)
C3—C8—C7	101.79 (12)	C15—C20—H20	120.4
C9—C8—H8	112.3	C19—C20—H20	120.4
C3—C8—H8	112.3	C7—O11—C4	97.80 (11)
C7—C8—H8	112.3	C2—N1—C9	113.03 (12)
O14—C9—N1	123.91 (14)	C2—N1—C15	123.87 (12)
O14—C9—C8	127.69 (14)	C9—N1—C15	123.07 (13)
			
O13—C2—C3—C8	−177.05 (16)	C3—C8—C9—N1	1.13 (16)
N1—C2—C3—C8	2.91 (16)	C7—C8—C9—N1	111.06 (14)
O13—C2—C3—C4	72.9 (2)	C20—C15—C16—C17	0.4 (2)
N1—C2—C3—C4	−107.14 (14)	N1—C15—C16—C17	−179.47 (15)
C2—C3—C4—O11	78.04 (14)	C15—C16—C17—C18	0.2 (3)
C8—C3—C4—O11	−34.09 (12)	C16—C17—C18—C19	−0.3 (3)
C2—C3—C4—C10	−43.66 (18)	C17—C18—C19—C20	−0.2 (3)
C8—C3—C4—C10	−155.80 (13)	C16—C15—C20—C19	−0.8 (2)
C2—C3—C4—C5	−177.18 (13)	N1—C15—C20—C19	179.01 (14)
C8—C3—C4—C5	70.69 (14)	C18—C19—C20—C15	0.7 (2)
O11—C4—C5—C6	30.57 (17)	C12—C7—O11—C4	173.54 (12)
C10—C4—C5—C6	153.10 (16)	C6—C7—O11—C4	47.34 (13)
C3—C4—C5—C6	−72.78 (16)	C8—C7—O11—C4	−60.23 (12)
C4—C5—C6—C7	−0.22 (19)	C10—C4—O11—C7	−174.15 (12)
C5—C6—C7—O11	−30.23 (17)	C5—C4—O11—C7	−47.72 (13)
C5—C6—C7—C12	−152.76 (16)	C3—C4—O11—C7	59.33 (12)
C5—C6—C7—C8	72.58 (17)	O13—C2—N1—C9	177.59 (15)
C2—C3—C8—C9	−2.40 (16)	C3—C2—N1—C9	−2.37 (18)
C4—C3—C8—C9	115.90 (12)	O13—C2—N1—C15	−0.8 (3)
C2—C3—C8—C7	−119.87 (12)	C3—C2—N1—C15	179.28 (13)
C4—C3—C8—C7	−1.57 (13)	O14—C9—N1—C2	179.87 (15)
O11—C7—C8—C9	−75.16 (14)	C8—C9—N1—C2	0.75 (18)
C12—C7—C8—C9	46.41 (18)	O14—C9—N1—C15	−1.8 (2)
C6—C7—C8—C9	−179.58 (12)	C8—C9—N1—C15	179.11 (13)
O11—C7—C8—C3	36.83 (13)	C20—C15—N1—C2	118.53 (17)
C12—C7—C8—C3	158.40 (13)	C16—C15—N1—C2	−61.6 (2)
C6—C7—C8—C3	−67.59 (14)	C20—C15—N1—C9	−59.6 (2)
C3—C8—C9—O14	−177.95 (16)	C16—C15—N1—C9	120.18 (17)
C7—C8—C9—O14	−68.0 (2)		

### Hydrogen-bond geometry (Å, º)

Cg1 is the centroid of the C15–C20 phenyl ring.

**Table:**

*D*—H···*A*	*D*—H	H···*A*	*D*···*A*	*D*—H···*A*
C5—H5···O14^i^	0.93	2.57	3.362 (2)	144
C10—H10*B*···*Cg*1^i^	0.96	2.95	3.8029	148
C12—H12*B*···*Cg*1^ii^	0.96	2.79	3.7287	167

Symmetry codes: (i) *x*−1, *y*, *z*; (ii) *x*, −*y*−1/2, *z*−1/2.
